# Advanced Hydrogels as Wound Dressings

**DOI:** 10.3390/biom10081169

**Published:** 2020-08-11

**Authors:** Shima Tavakoli, Agnes S. Klar

**Affiliations:** 1 Department of Materials Engineering, Isfahan University of Technology, Isfahan 84156-83111, Iran; Shimatavakoli1995@gmail.com; 2 Tissue Biology Research Unit, University Children’s Hospital Zurich, University of Zurich, 75, 8032 Zurich, Switzerland; 3 Children’s Research Center, University Children’s Hospital Zurich, 75, 8032 Zurich, Switzerland

**Keywords:** tissue engineering, skin substitutes, sprayable “smart” hydrogels, “in situ” forming hydrogels, wound dressings with integrated sensors, regenerative medicine

## Abstract

Skin is the largest organ of the human body, protecting it against the external environment. Despite high self-regeneration potential, severe skin defects will not heal spontaneously and need to be covered by skin substitutes. Tremendous progress has been made in the field of skin tissue engineering, in recent years, to develop new skin substitutes. Among them, hydrogels are one of the candidates with most potential to mimic the native skin microenvironment, due to their porous and hydrated molecular structure. They can be applied as a permanent or temporary dressing for different wounds to support the regeneration and healing of the injured epidermis, dermis, or both. Based on the material used for their fabrication, hydrogels can be subdivided into two main groups—natural and synthetic. Moreover, hydrogels can be reinforced by incorporating nanoparticles to obtain “in situ” hybrid hydrogels, showing superior properties and tailored functionality. In addition, different sensors can be embedded in hydrogel wound dressings to provide real-time information about the wound environment. This review focuses on the most recent developments in the field of hydrogel-based skin substitutes for skin replacement. In particular, we discuss the synthesis, fabrication, and biomedical application of novel “smart” hydrogels.

## 1. Introduction

Skin is the largest organ of the body and serves as an important barrier, protecting the body from the external environment [1]. Although the human skin possesses high self-regeneration potential, skin defects measuring more than a certain diameter will not heal spontaneously and require skin transplants. Additionally, the wound healing process in some patients is impaired, leading to chronic wounds, which can eventually result in amputations or even mortality [2].

Today’s “gold standard” treatment methods are primarily split- and full-thickness skin grafts, as well as skin flaps, skin expansion techniques, and dermal substitutes [3,4,5]. However, serious problems associated with the aforementioned methods are typically donor site shortage and hypertrophic scars or keloids, which eventually lead to severe functional and psychosocial problems [6,7]. Therefore, tissue-engineered skin substitutes offer a promising alternative to overcome these limitations.

Skin tissue engineering is a rapidly growing field, with the aim to develop skin substitutes for clinical applications [8,9,10]. Those skin substitutes represent a heterogeneous group of wound dressing materials that can be placed on the wound site to replace the functions of the skin, either temporarily or permanently, depending on the product features [11].

There are various materials, namely natural or synthetic, which have been employed in skin substitutes [12]. Among them, hydrogel-based skin substitutes have attracted intense attention due to their unique characteristics to mimic the native skin microenvironment in comparison with other materials. Additionally, hydrogels can be applied as sprayable wound dressings and can be reinforced by incorporating nanoparticles to obtain “in situ” forming nanocomposite hydrogels, also known as “smart” hybrid hydrogels, which can have superior properties and tailored functionality [13,14,15].

The current review summarizes the progress of hydrogels in the field of skin tissue engineering and provides an overview of the developments as well as advantages and shortcomings of various commercially available hydrogel-based skin substitutes and wound dressings.

## 2. Skin Structure

**Figure 1:**
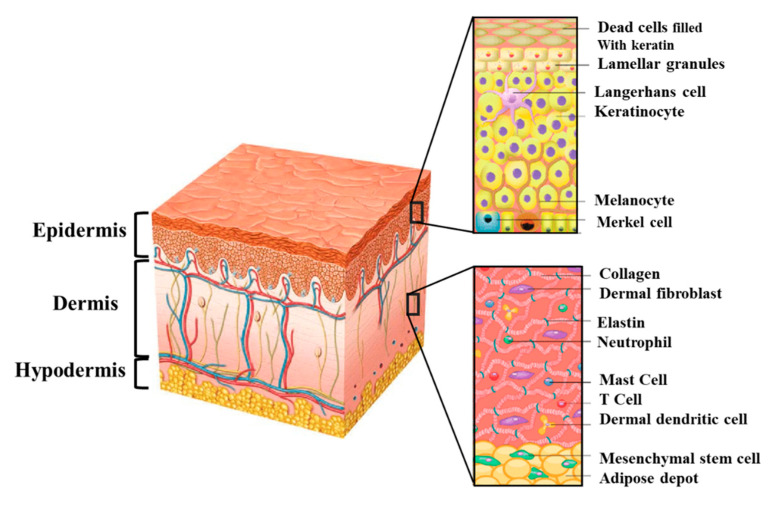
The structure of human skin consisting of three primary layers—the epidermis, the dermis, and the hypodermis. The two inserts (right site) show their detailed cellular structure at higher magnification. The epidermis consists mainly of keratinocytes, melanocytes, and Langerhans cells. The dermis contains fibroblasts, neutrophils, mast cells, and dermal dendritic cells embedded within the dermal matrix rich in collagen and elastin. Beneath the dermis lies the subcutaneous adipose tissue (hypodermis) containing mesenchymal stem cells.

Skin is the largest organ of the human body and acts as the first line of defense from external physical, chemical, and biological factors [1]. Moreover, skin performs many vital functions, including prevention of water loss from the body, and it has a role in body temperature regulation. Normal human skin consists of three layers—the epidermis, the dermis, and the hypodermis (Figure 1). The barrier function of the skin is provided by the epidermis, which is composed mainly of keratinocytes. They form a stratified epithelium, with basal keratinocytes at the innermost layer and the keratinized, relatively impermeable outer stratum corneum layer on the surface [16]. Other epidermal cells include melanocytes that provide skin pigmentation; Langerhans cells that are antigen-presenting dendritic cells of immune system; Merkel cells that are thought to function as mechanoreceptors forming close contacts with sensory neurons [17]. The epidermis is constantly renewing, with the basal keratinocytes undergoing continuous proliferation to rebuild the entire epidermis [18]. Distinct basal stem cells divide, yielding a subset of daughter cells that undergo a cell cycle arrest and start to migrate upward towards the surface of the skin. This migration generates four different layers of the epidermis; the inner most layer is the basal layer (stratum basale), which is followed by the spinous or prickle cell layer (stratum spinosum), granular layer (stratum granulosum), and the cornified layer (stratum corneum) as the outer most layer [19]. In this process, called terminal differentiation, keratinocytes eventually lose their nuclei and undergo programmed cell death.

The epidermis is separated from the dermis by the so called dermal–epidermal junction. This junction is formed by the basement membrane, which anchors keratinocytes in the epidermis. The basement membrane is a specialized extracellular matrix (ECM) structure containing different proteins such as laminin, nidogen, collagen types IV and VII, and the proteoglycans, perlecan, and collagen XVIII. In addition, it also contains collagen type I and III, tenascin, and fibrillin-1 [20,21]. Both keratinocytes present in the epidermis and fibroblasts from the underlying dermis contribute to the formation of the basement membrane [22,23,24].

The dermis is located beneath the epidermis and is the thickest layer of the skin (1.5 to 4 mm thick). Fibroblasts represent the main cell type in the dermis and synthesize collagen and elastin to provide mechanical strength and elasticity. The other main cells in the dermis include human dermal microvascular endothelial cells (HDMECs), pericytes, and mast cells. Further, the dermis harbors different structures such as sweat glands along with hair follicles, sebaceous glands, and nerve endings. Moreover, the dermis contains a rich network of nerves, blood, and lymphatic vessels. The dermis is organized into two layers—the papillary and the reticular dermis. The superficial papillary dermis is composed of loose connective tissue and contains numerous blood vessels. In contrast, the reticular layer is located underneath the papillary dermis and contains thick collagen and elastin fibers. Those collagen fibers build a thick layer of connective tissue [25].

Beneath the dermis lies the subcutaneous adipose tissue called hypodermis, which is composed primarily of fat and connective tissue. The hypodermis reduces heat loss by providing thermal insulation and cushioning between the skin and skeletal structures, such as bone and muscle [26]. The hypodermis is rich in blood and lymph vessels that run through it. Further perspiratory glands and hair follicles reach into it. In addition, the hypodermis is considered an endocrine organ that serves as an energy storage area [1,27].

Normal skin is lubricated and slightly acidic, which protects it from the growth of pathogens. Furthermore, Langerhans cells, which are present in the epidermis, also protect from infections [28,29].

## 3. Skin Wound Healing

**Figure 2:**
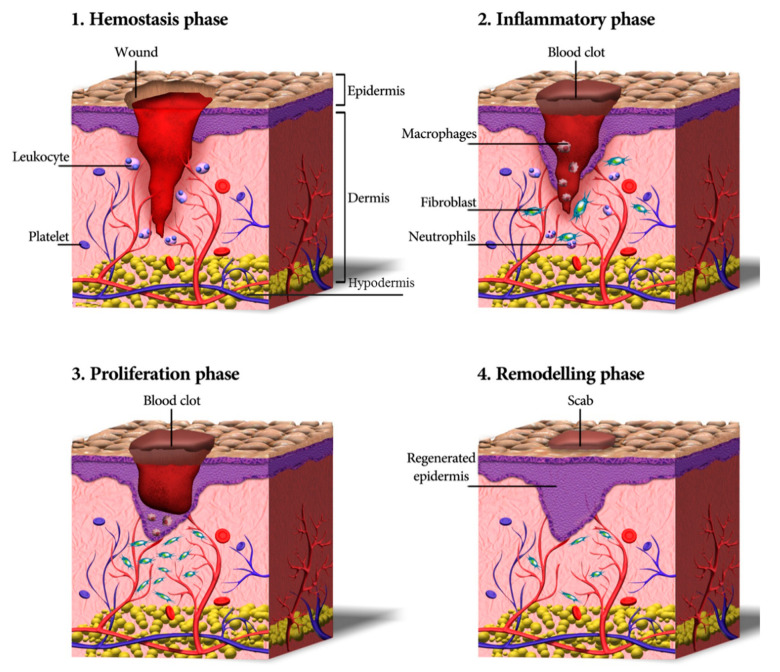
A schematic depicting the process of wound healing, including four continuous phases—homeostasis, inflammation, proliferation, and remodeling. In these four overlapping stages. First, blood platelets are activated to form a blood clot and have also a role in leukocyte recruitment. Next, neutrophils and macrophages clean the wound site from dead cells, bacteria, and other pathogens or debris. Then, fibroblasts migrate, proliferate, and activate the angiogenesis process. Finally, granulation tissue is formed, the extracellular matrix proteins are deposited to reconstitute the dermal tissue, and the epidermis is regenerated. Eventually, many of the formed capillaries and fibroblasts disappear.

Wound healing is a dynamic and complex process that can be divided into four subsequent and overlapping phases—homeostasis (blood clotting), inflammation, tissue growth (proliferation), and tissue remodeling (maturation) (Figure 2) [30,31]. Within the first few minutes after injury, blood platelets start to stick to one another and to the wound site. In contact with collagen, platelets change into an amorphous shape, resulting in their activation and aggregation. Further, thrombin starts to be produced and catalyzes the initiation of the coagulation cascade [32,33]. This, in turn, results in the activation of fibrin, which forms a mesh preventing further bleeding [34]. Moreover, platelets have a crucial role in leukocyte recruitment and the initiation and progress of inflammation [35].

In the inflammatory phase, immune cells (particularly neutrophils and macrophages) are recruited into the wound, where they phagocyte damaged and dead cells, bacteria, and other pathogens or debris [26]. Moreover, inflammatory cells together with platelets release various peptide growth factors, promoting the migration of fibroblasts into the wound site and activating angiogenesis [36]. During the proliferation phase, fibroblasts are further stimulated to proliferate in the wound area. Further, they reconstitute the dermal tissue components by formation of granulation tissue and deposition of extracellular matrix proteins, mainly collagen [37,38]. Furthermore, enhanced angiogenesis induces ingrowth of a new network of blood vessels into the granulation tissue to enhance cell survival by providing sufficient levels of oxygen and nutrients. Afterward, epithelial cells migrate from the wound edges to cover the defect, a process known as ‘epithelialization’.

Finally, during wound remodeling, the excess collagen fibers are degraded in the dermis, and wound contraction begins to peak. The healed wound reaches its maximum mechanical. The ultimate resulting scar will have 80% of the original strength of the wound [39].

## 4. Types of Wounds

Wounds are injuries that break the skin, leading to disruption of its normal anatomic structure and function [40]. There are different types of wounds, which can be caused by physical, chemical, and thermal damages. Based on the nature of the repair process, wounds can be divided into two main categories—acute and chronic wounds. Acute wounds are injuries that are healed completely, with minimal scarring and within the period of approximately 8–12 weeks. These types of wounds are mostly caused by mechanical injuries, like frictional contact between the skin and hard surfaces (e.g., knives), gun shots penetration, and surgical incisions. In contrast, acute wounds result mainly from chemical and burn injuries after radiation, corrosive chemicals, electricity, and thermal injuries [41]. On the other hand, chronic wounds are defined as wounds that show delayed healing 12 weeks after initial injury [40]. Those wounds are mostly caused by repeated tissue insults or underlying physiological conditions like diabetes, impaired angiogenesis and innervation or cellular migration [42]. Other reasons might be malignancies, infections, poor primary treatment, and other patient related factors [43].

Based on causative etiologies, the Wound Healing Society divides chronic wounds into four distinct categories—pressure ulcers, diabetic ulcers, venous ulcers, and arterial insufficiency ulcers [40]. Although those different non-healing wounds may have different causes, they all share common wound characteristics, such as upregulated level of proteases, elevated pro-inflammatory cytokines, persistent reactive oxygen species (ROS), presence of senescent fibroblast, prolonged infection, as well as dysfunctional and insufficient stem cells [44].

## 5. Applications of Hydrogels for Wound Healing

Hydrogels represent a class of materials that are widely used in soft tissue engineering of skin, blood vessel, muscle, and fat [45]. Hydrogels are three-dimensional (3D) networks consisting of physically or chemically crosslinked bonds of hydrophilic polymers. The insoluble hydrophilic structures demonstrate a remarkable potential to absorb wound exudates and allows oxygen diffusion to accelerate healing [46,47].

Importantly, hydrogels possess a highly hydrated 3D polymeric network and can bind several-fold more water as compared to their dry weight and can thereby maintain a high moisture level of the wound bed. Due to these unique physical properties, hydrogel networks can be casted into various sizes and shapes [48,49]. Therefore, hydrogel-based materials are the most suitable dressings to cover skin wounds [50]. Furthermore, hydrogels offer a platform to load cells, antibacterial agents, growth factors, as well as distinct supplementary and biomacromolecules [51].

With regard to ECM similarity, hydrogels used for wound healing applications should provide a cell-friendly 3D environment to promote tissue regeneration, with or without the presence of cells embedded in the scaffold. Importantly, all hydrogels need to satisfy the basic requirements of biocompatibility in clinical use as well as possess unique physical and mechanical properties suited for skin wound applications [52,53]. Moreover, they also need to provide the appropriate microenvironment for vessel ingrowth and cellular proliferation.

Table 1 demonstrates some of the most important characteristics of wound dressings, which are absolutely necessary to support skin wound healing [54,55].

### 5.1. Sprayable “In Situ” Forming Hydrogels for Wound Applications

In recent years, “in situ” forming, so called “smart” wound dressings have been proposed to overcome key limitations of conventional dressings used for clinical applications. “Smart” hydrogels can be easily crosslinked by using different chemical or physical strategies such as photo-, thermo-, or ionic-crosslinking [56,57,58]. Among the different types of “smart” hydrogels, sprayable dressings are the most suitable “in situ” forming dressings to cover wounds. Those hydrogels exhibit numerous advantages, such as simple application without any specialist’s aid, patient satisfaction, and low production costs. Moreover, spray delivery can increase the penetration of the nanocomposite hydrogel into the wound area, thereby contributing to the improved delivery of active ingredients or therapeutic formulation to the wound [13,57,59]. However, the formulation of a sprayable wound dressing needs to be carefully adjusted to have an optimal viscosity to be applied as a spray-on dressing and cover the wound site uniformly [60]. Currently, several “in situ” forming sprayable hydrogels are available as wound dressings. Here, we describe recent advances, synthesis process, and biological applications of sprayable hydrogels used as wound dressings, with a particular focus on the use of natural hydrogels.

#### 5.1.1. Methacrylated Gelatin

Gelatin is a type of natural hydrophilic polymer obtained after hydrolysis and denaturation of collagen under high temperature. Gelatin has some advantages as a wound dressing, including high biocompatibility, solubility, degradability, easy extraction, and synthesis [61,62]. Moreover, gelatin polymers mimic, to some extent, the natural dermal extracellular matrix and can be therefore easily employed for wound dressings. For example, in a study by Neumann et al. [63], a gelatin-based spray-on foam bandage for use on skin wounds was developed. The aqueous gel is first sprayed on the wound site from aerosol containers and effectively covers the uneven wound surface. Subsequently, the sprayed foam dries and adheres to the wound, providing a three-dimensional matrix, which reduces evaporative water loss. Moreover, this foam also possesses antimicrobial activity against Gram-positive, Gram-negative, and fungal contaminants [63].

**Figure 3:**
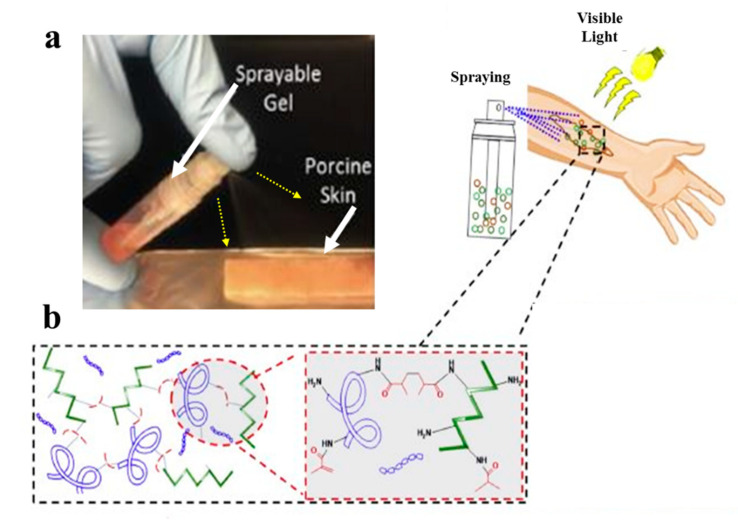
(**a**) Spraying of the MeTro/GelMA composite hydrogel on a porcine skin and the crosslinking process of the MeTro/GelMA under visible light exposure; (**b**) MeTro/GelMA monomers and their bonds under light exposure [65].

However, gelatin is characterized by insufficient and uncontrollable mechanical properties and high degradation rate for wound healing applications. To overcome these limitations, specific modifications of gelatin such as its methacrylation or blending with other hydrogels, may be applied [64]. Accordingly, Annabi et al. [65] employed methacrylated gelatin (GelMA) and methacryloyl-substituted recombinant human tropoelastin (MeTro) to synthesize a sprayable MeTro/GelMA composite hydrogel. The authors demonstrated that this sprayable hydrogel can be efficiently crosslinked under visible light exposure. Figure 3 demonstrates spraying of the MeTro/GelMA composite hydrogel over porcine skin and the interactions between monomers with methacrylated groups under light exposure.

#### 5.1.2. Methacrylated Kappa-Carrageenan

Methacrylated kappa-carrageenan (KaMA) is a hydrogel based on kappa-carrageenan (κCA), a natural linear water-soluble polysaccharide with one sulfated group per disaccharide (25 to 30% ester sulfate content) [52]. κCA resembles natural glycosaminoglycan structure and, hence, provides similar physical and mechanical properties to native human skin [66]. Importantly, the KaMA hydrogel can be crosslinked chemically under visible light exposure and physically by ion interactions. Recently, Mihaila et al. [57] introduced a dual-crosslinkable hydrogel based on KaMA for tissue engineering applications using potassium ions and UV light exposure. The authors used KaMA with different methacrylation degrees (low, medium, and high) and compared distinct mechanical properties of those hydrogels. n another study, Mokhtari et al. [67] reported the addition of dopamine functionalized graphene oxide to the dual-crosslinkable KaMA hydrogel network. This modification eventually resulted in increased shear-thinning behavior, which, in turn, improved the injection and spraying abilities of the hydrogel. However, Mokhtari et al. and Mihaila et al. used UV light in their studies, it can cause cell death, DNA damage, immunosuppression, cancer, and accelerating tissue aging [65].

**Figure 4:**
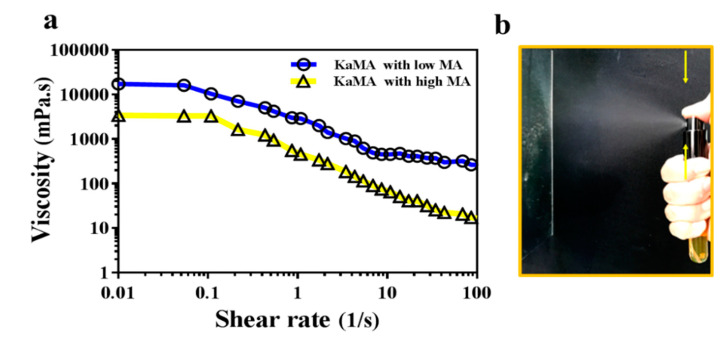
(**a**) Rheological evaluation of KaMA hydrogel with low and high MA degree; as shear rate increases, the viscosity drops; (**b**) spraying ability of the KaMA hydrogel with high MA [60].

Therefore, in a recent study, we optimized this method, using a dual-crosslinking KaMA hydrogel prepared under visible light exposure and ion interaction [60]. Furthermore, we demonstrated that the methacrylation degree (MA) and polymer concentration improved the shear-thinning behavior and viscosity of the KaMA solution. This resulted in excellent spraying properties of KaMA, as confirmed by rheological tests (Figure 4) [60]. Generally, spray- or inject-ability of hydrogels could be facilitated with lower viscosity of primary solution due to the reduction in droplet size after pumping, which can vary based on MA degree and polymer concentration [58,60].

**Figure 5:**
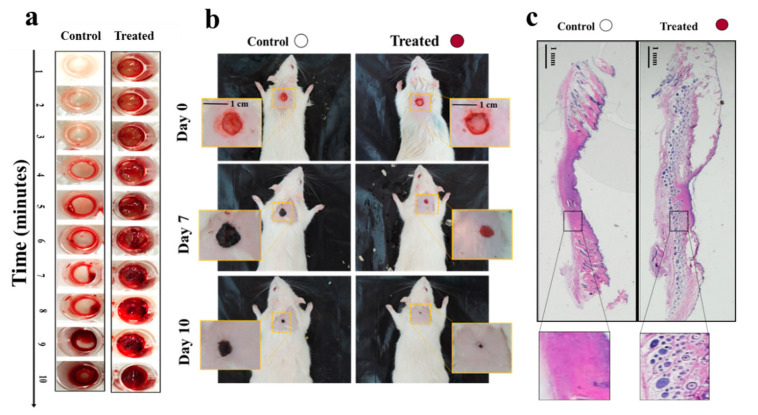
(**a**) Comparison of blood clot formation time in vitro between the control surface (surface without hydrogel) and the treated surface, containing the nanocomposite hydrogel with ZnO/PD and L-glutamic acid; (**b**) differences in wound size between control (untreated) wounds and wounds covered with the nanocomposite hydrogel containing ZnO/PD and L-glutamic acid in vivo; (**c**) histological analyses comparing the appearance of control wounds versus wounds treated with the nanocomposite hydrogel with ZnO/PD and L-glutamic acid in vivo [52].

In another study, we proposed a “smart in situ” forming nanocomposite hydrogel dressing based on polydopamine modified ZnO (ZnO/PD) in KaMA matrix for diabetic wounds [52]. In this study, a KaMA hydrogel containing ZnO/PD nanoparticles was crosslinked solely by exposure to visible light and evaluated thereafter. In addition, L-glutamic acid was added to the KaMA hydrogel matrix to accelerate wound contraction. Our results revealed that ZnO/PD and L-glutamic acid could be efficiently encapsulated in the hydrogel network with a controllable release profile. Eventually, in vitro and in vivo tests demonstrated that the nanocomposite KaMA hydrogel spray containing ZnO/PD and L-glutamic acid proved to be highly effective as hemostatic and antibacterial dressing, thus, facilitating a rapid wound closure (Figure 5).

#### 5.1.3. Chitosan

Chitosan is a natural cationic copolymer with hydrophilic properties, that can be degraded by human enzymes, thus, showing high biocompatibility and biodegradability [68,69]. Moreover, hitosan-based hydrogels demonstrate excellent bioadhesive, bacteriostatic, and hemostatic properties, with the ability to bind with red blood cells, causing blood to clot [70,71]. These unique properties make chitosan a promising biopolymer for tissue engineering purposes. In the study of Gholizadeh et al. [72], a sprayable thermo-crosslinkable hydrogel based on chitosan biopolymer was tested for a local treatment of nasal wounds. The authors demonstrated that the developed formulation can undergo a rapid liquid-to-gel phase change within approximately 5 min at 32 °C, which is proportional to the human nasal cavity temperature range [72]. Another study performed by Mattioli-Belmonte et al. [73] revealed the effectiveness of chitin nanofibril/chitosan glycolate-based hydrogels for healing of skin lesions [73]. This sprayable hydrogel was compared with two other forms of dressings with similar components, including a gel and a gauze form. Interestingly, the results of this study indicated that the sprayable hydrogel form seems to be a more effective treatment option in healing of superficial skin lesions.

LQD (Brancaster Pharma) wound spray is a new spray-on dressing that contains chitosan FH02™, a unique form of chitosan in aqueous solution. This sprayable wound dressing is primarily indicated for the local treatment of chronic wounds, such as leg ulcers and diabetic foot ulcers, but also for acute wounds and epidermal and superficial partial-thickness burns [74]. Several clinicians have evaluated this product in various clinical settings and confirmed its easy and safe application [74,75]. Moreover, LQD wound spray shows some antibacterial and hemostatic properties due to the presence of chitosan [76].

### 5.2. Acellular Hydrogel-Based Wound Dressings

There are also conventional wound dressings based on hydrogels which can be produced as films or sheets. Hydrogel wound dressing films can be synthesized from natural or synthetic crosslinked polymers. There are various types of hydrogel dressings, based either on synthetic (poly (methacrylates), poly-vinylpyrrolidine, polyvinyl alcohol, and polyurethane) or natural components [77]. Recently, a comprehensive review article by Cascone et al. [78] summarized acellular hydrogel-based commercial wound dressings for biomedical applications. Table 2 summarizes the recent and most frequently used commercial acellular hydrogel-based wound dressings, along with their main components and applications [78].

#### 5.2.1. Commercial Dermal Substitutes Based on Natural Hydrogels

Natural hydrogels are frequently used as dermal skin substitutes due to their unique properties such as high water content, softness, flexibility, and biocompatibility [77,79]. Therefore, there are several dermal substitutes on the market that are based on natural hydrogel components.

The Integra dermal regeneration template is one of many off-the-shelf acellular products for the regeneration of dermal tissue [80]. Integra artificial dermis is manufactured as a biosynthetic template composed of a porous matrix of crosslinked bovine tendon collagen and glycosaminoglycan, and is covered with a semi-permeable polysiloxane (silicone) layer. This silicone membrane controls water vapor loss, provides a flexible adherent covering for the wound surface, and adds increased tear strength to the template [81]. Importantly, the collagen–glycosaminoglycan biodegradable matrix of the Integra enables sufficient ingrowth of host blood vessels and fibroblasts, and provides immediate wound closure and permanent regeneration of the dermis. The Integra is applied on a debrided wound bed, especially in burn injuries [82,83]. In particular, it is indicated for use in partial- and full-thickness wounds, pressure ulcers, venous ulcers, diabetic ulcers, chronic vascular ulcers, as well as surgical, traumatic, and draining wounds [84,85]. First, the membrane-covered Integra is placed on the wound usually for 3–6 weeks and allowed to be invaded by fibroblasts and vascularize, before adding keratinocytes. Once the neodermis is formed, the silicone membrane is removed, and autologous split-thickness skin can be applied over the neodermis [3,86].

Another dermal substitute, Matriderm, is based on a highly porous membrane consisting of bovine type I collagen and elastin [87]. The collagen used for the matrix is extracted from bovine dermis, and elastin is derived from bovine nuchal ligament by hydrolysis. Matriderm is employed for dermal regeneration, especially in burn and chronic wounds [88,89]. Due to its hemostatic properties, Matriderm was also reported to reduce the risk of hematoma formation, which often occurs after skin grafting [90]. There are several reports of effective skin engraftment using Matriderm in a one-step procedure in the clinics [91,92]. Furthermore, one study compared the effectiveness of using Matriderm versus Integra in full-thickness skin defects of immune-incompetent rats [93]. Interestingly, although the structure, composition, and crosslinking of these two dermal substitutes are different, the results of this study revealed no significant differences between Matriderm and Integra regarding neodermis formation or vascularization potential, and acceptance of grafts. Moreover, the crosslinking of Integra with glutaraldehyde results in higher resistance of Integra to degradation in comparison with Matriderm, which lacks any crosslinking in its preparation [93].

Overall, naturally derived skin substitutes frequently have superior biocompatibility over synthetic polymers. However, it must be noted that they are prone to some batch-to-batch variations, leading to unstable physical and chemical features in some cases [94]. Moreover, mechanical properties of skin substitutes derived from natural scaffolds are often poor and need to be strengthened by biodegradable meshes [95].

#### 5.2.2. Commercial Dermal Substitutes Based on Synthetic Hydrogels

Synthetic hydrogels used as skin templates represent some advantages as compared to naturally derived polymers [96]. First, they demonstrate predictable and controllable characteristics, such as easy shape control, low production costs, and stable mechanical properties. Second, synthetic scaffolds are convenient to produce in terms of their stable formulation. However, synthetic materials should be selected precisely to exhibit low risk of transplant rejection and disease transmission for bioapplications [97].

Polyvinyl alcohol (PVA) is one of the most frequently used synthetic polymers employed as a dermal wound dressing [98]. PVA hydrogels are often used in combination with other polysaccharide-based hydrogels, such as starch, alginate, and carrageenan, due to its insufficient elasticity, membrane stiffness, and inadequate hydrophilic properties [79,99,100].

Polyurethane (PU) is another synthetic polymer frequently used in various commercial wound dressings. One example is the NovoSorb biodegradable temporizing matrix (BTM). This is a fully synthetic temporary dermal replacement template based on a biodegradable PU foam, allowing for the infiltration of cells and serving as a matrix for the reconstruction of deeper layers (dermis) of the skin. The PU foam is covered by a non-biodegradable PU seal designed to physiologically close the wound and limit evaporative moisture loss [101]. The BTM is applied particularly in deep burns, when split thickness skin grafts alone are insufficient for operative tissue reconstruction [102]. Moreover, the BTM was already successfully used for the reconstruction of defects following serial debridement of necrotizing fasciitis, which are associated with a significant mortality rate [102]. Moreover, the application of the BTM substitute has been also reported for the reconstruction, following radical debridement and necrotizing fasciitis wounds, which are often deep and poorly vascularized [103,104]. Interestingly, these studies revealed that the PU structure of the BTM was not affected by the underlying wound infection. Moreover, the authors demonstrated that the BTM continued to integrate into the wound bed following drainage of infected collections through perforations in the seal. This, in turn, resulted in improved healing of those deep and extensive wounds [105,106]. In addition, the BTM has been also used for the reconstruction of soft tissue defects, such as on the anterior neck, lateral chest and flank, and knee from mid-thigh to distal leg [106].

### 5.3. Hydrogel Dressings with Integrated Sensors

Nowadays, hydrogel wound dressings are employed to protect and seal injury site. In addition, many of them have been designed to release drugs or compounds in a controllable manner to prevent infection and accelerate the wound healing process [107,108]. However, they are incapable of providing reliable information regarding general healing status, like its bacterial density, oxygenation amount, inflammation level, temperature, and pH [107]. These limitations inspired the development of sensor-based wound dressings, which can provide important information about conditions of the wound. These kinds of wound dressings offer several advantages, including improved wound care treatment, reduced hospitalization time and healthcare costs, and reduction in wound dressing exchanges [109].

Recently, various sensors have been developed to measure different biomarkers such as pH, temperature, moisture, oxygen of wound, mechanical and electrical properties of skin or wound, and upregulation or downregulation of enzyme levels [107]. Importantly, all of the sensors should have some essential characteristics, including proportional flexibility to the hydrogel film and to body contours, biocompatibility, and non-toxicity to the immune system. Moreover, it is critical that such sensors are resistant to wound exudate. Additionally, for degradable wound dressings, the integrated sensors should be degraded with a proportional degradation rate to the hydrogel matrix rate and with non-toxic degradation debris to the immune system [107,110,111]. Moreover, it is vitally important to design novel dressings with integrated sensors, which are able to monitor the early status of the wound [112]. Among different biomarkers, temperature is considered as one of the most promising indicators for an early detection of the inflammation and infection in the wound bed; indeed, abnormal wound temperature variations can be selected as an early predictor of infection before any other symptom emergence [113]. On the other hand, it is also important to select appropriate sensors to measure a particular biomarker based on the wound type [114]. For this, however, the integration of more than one sensor into a wound dressing is needed. This approach can provide even more accurate information about wound status. However, such multitude sensors-based dressings need a precise design and are, therefore, more expensive.

**Figure 6:**
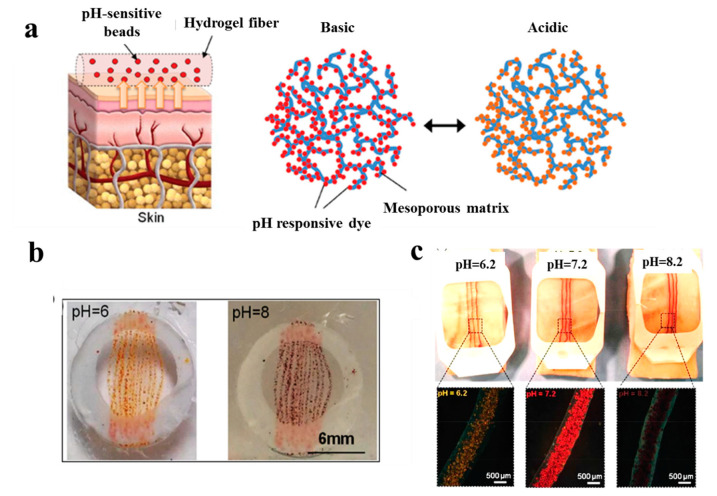
(**a**) Schematic illustrating the application of a pH sensor-based hydrogel containing pH-sensitive beads on skin and the color changes in basic and acidic environment; (**b**) macroscopic pictures of hydrogel fiber-based patches with pH-sensitive beads under acidic and basic conditions; (**c**) images representing color changes of pH-sensitive dressings placed on pig skin when sprayed with solutions of different pH values [115].

In a study performed by Tamayol et al. [115], pH-responsive alginate-based hydrogel microfibers were used for long-term monitoring of the epidermal wound environment. First, mesoporous microparticles of polyester beads were loaded with a pH sensitive dye, and then, embedded in the alginate hydrogel microfibers by using a microfluidic spinning method for fabricating fibers with diverse shapes and sizes. The pH of wounds is a critical parameter related to angiogenesis, protease activity, and bacterial infection [116]. For example, chronic non-healing wounds are known to have a high alkaline environment, while the healing process occurs more efficiently in an acidic condition [117]. Thus, fabricated dermal patches capable of continuous pH measurement of the wound are crucial to monitor the healing process and guide the point-of-care treatment to finally improve chronic wound therapy outcome. Figure 6 demonstrates colorimetric pH sensor-based hydrogels carrying beads loaded with pH-sensitive dye and its application on a wound. Importantly, the color of this wound dressing changes rapidly under acidic or basic environments, slowing for long-term monitoring of wound.

**Figure 7:**
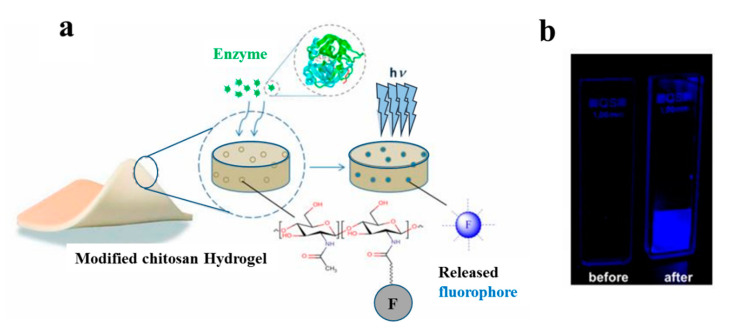
(**a**) Schematic of the interaction between modified chitosan hydrogel and an enzyme; (**b**) images of modified hydrogel before and after enzymatic degradation [118].

In another study, a hydrogel-based dressing for the detection of enzymes was applied and studied in infection-sensing wound dressings [118]. In this regard, a modified chitosan hydrogel was functionalized with a fluorogenic substrate, which is released by enzymatic degradation (Figure 7). This model is capable of detecting the presence of different types of enzymes by using appropriate fluorogenic or chromogenic substrates. Moreover, it is highly useful for the detection of specific pathogenic bacteria in wound dressings.

Occhiuzzi et al. [119] worked on a hydrogel membrane including a radio-frequency identification (RFID) epidermal sensor for monitoring wound conditions. This hydrogel membrane was synthesized based on a polyvinyl alcohol/xyloglucan (PVA/XG) hydrogel, which is able to absorb wound exudates and release water and drugs or biomolecules (e.g., antibacterial agents or growth factors), thus, providing appropriate conditions for the wound healing process. In addition, the epidermal sensor was capable of measuring the local temperature and could, thus, provide meaningful biological information about the status of the wound.

**Figure 8:**
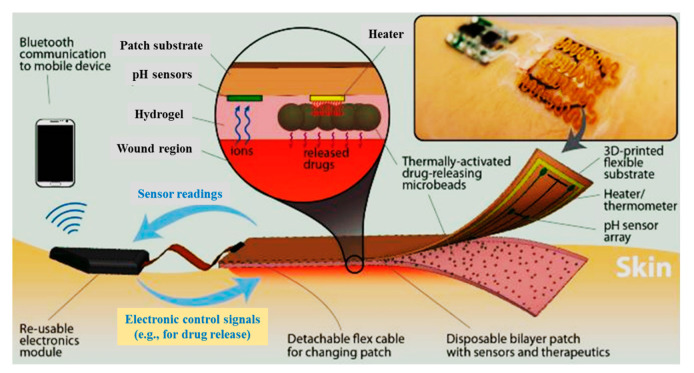
Schematic of a flexible “smart” wound dressing. The dressing is comprised of a pH sensor and a flexible heater to trigger thermo-responsive carriers containing drugs. Drug carriers are embedded in a sheet of alginate hydrogel, casted around the pH sensors and on the heater. Finally, the sensors and the heater are connected to an electronic module that is able to record the data from sensors and power the heater. The electronic module can also communicate with computers and smartphones wirelessly [120].

Moreover, an automated flexible wound dressing for the monitoring and treatment of chronic wounds was developed by Mostafalu et al. [120]. This dressing contains numerous components, including a patch with flexible temperature and pH sensors, a hydrogel release sheet, thermo-responsive drug-loaded carriers with an integrated microheater, and an electronic patch (Figure 8). First, potentiometric pH and temperature sensors provide information about bacterial infection and inflammation level, respectively. Afterwards, thermo-responsive drug carriers provide a controllable drug release system in response to temperature changes. The drug-carriers embedded within an alginate hydrogel patch are placed on top of a flexible heater, and then, the entire system is attached to a transparent medical tape to form a wearable matrix. Generally, this sophisticated approach allows for monitoring of pH and temperature in real-time with on-demand drug release. This flexible smart wound dressing has the potential to significantly impact the treatment of chronic wounds in the future.

In general, smart wound dressing could revolutionize wound care quality and can have a major effect on therapeutic outcomes. They can solve many of the challenges associated with wound healing, in particular with chronic wounds, by allowing sensing, responding, and reporting information of the wound environment in real-time. Thus, smart wound dressing can improve wound management and long-term clinical outcomes. Moreover, sensors with integrated active drug delivery systems can rapidly respond to potential infections or hyper-inflammation in chronic wounds [107].

## 6. Conclusions and Future Direction

Considerable progress has been made over recent years in the field of skin replacement therapies, with various natural and synthetic biomaterials being used for fabrication of skin substitutes. Among those different scaffolds, hydrogels are widely used as wound dressings as they can better mimic the biological properties of human skin. Moreover, hydrogels are available in various forms, such as film, sprayable, and injectable gels. In addition, there are “smart” hydrogel-based wound dressings with integrated sensors that deliver real-time information about the status of the wound healing process.

Recently, sprayable hydrogel-based wound dressings have emerged as convenient scaffolds for wound care due to their versatile properties. Numerous research studies also indicate significant and growing interest in the synthesis and fabrication of such hydrogels and developing new “in situ” forming “smart” nanocomposite hydrogels for various biomedical applications. Additionally, understanding and regulating the interactions between polymeric chains and cells will inspire future studies of nanocomposite hydrogels. Moreover, new fabrication technologies will further allow the development of attractive scaffolding materials with an improved cellular microenvironment mimicking native human skin tissue. Therefore, the next generation of wound healing therapy will most likely focus on cell-laden “smart” nanocomposite-based hydrogels with integrated sensors to treat non-healing wounds.

## Figures and Tables

**Table 1:** Characteristics of an ideal wound dressing.

Feature	Description
No toxic component	Free from toxic materials that can damage and lead to dire consequences
Prevention of bacterial infection	Preventing bacterial infections, which could impair wound healing and prolong its duration
Adhesiveness	Providing an optimum amount of adhesive material to the wound site (excessive adhesive sustains an injury)
Moisture	Maintaining the optimum moisture level to promote cell migration and proliferation
Thermal insulation	Maintaining the optimal temperature of wound site to reduce pain
Absorption of excess exudate	Regulating the amount of exudates present in the wound
Oxygen permeability	Allowing the diffusion of oxygen to the wound bed to accelerate cell activity
Mechanical and physical properties	Resembling the structure of native skin
Minimal tissue trauma (pain)	Minimizing patient pain during application and removal
Cost effectiveness	Providing an affordable wound dressing
Free availability	Accessible for all patients or healthcare centers

**Table 2:** Some examples of commercial hydrogel-based wound dressings [78].

Product	Company	Main Component	Application
**Algisite**	Smith and Nephew	Alginate	Lacerations, abrasions, skin tears and minor burn wounds
**Medihoney**	Derma Sciences	Alginate	Partial to full-thickness wounds and burns
**Kaltostat**	Convatec	Alginate	Moderate to heavily exuding wounds chronic and acute wounds
**NU-GEL**	Systagenix	Alginate	Management of chronic wounds throughout all stages of healing
**Condress**	Smith and Nephew	Collagen	Chronic and acute wounds
**Helix3-cm**	Amerx Health Care	Collagen	Chronic and acute wounds
**DermaFilm**	DermaRite	Hydrocolloids	Abrasions, closed surgical wounds, superficial ulcers, and skin grafts
**Comfeel**	Coloplast	Hydrocolloids	Designed for difficult-to-dress areas
**CovaWound**	Covalon	Hydrocolloids	Pressure, leg and foot ulcers, superficial partial-thickness burns
**Inadine**	Systagenix	Polyethylene glycol	Open wounds that may become infected
**Sofargen**	Sofar	Colloidal silica	For abrasions, grazes, first- and second-degree burns and injuries
**Cutimed**	Bsn Medical	Dialkylcarbamoyl-chloride	Treatment of necrotic and sloughy tissues in chronic wounds
**Kendall**	Cardinal Health	Glycerin formulation	First- and second-degree burns and partial- and full-thickness wounds
